# Clinical Characteristics of Patients With Idiopathic Intracranial Hypertension in China

**DOI:** 10.7759/cureus.64990

**Published:** 2024-07-20

**Authors:** Tian Tian

**Affiliations:** 1 Department of Neurology, The First Affiliated Hospital of Zhengzhou University, Zhengzhou, CHN

**Keywords:** severe sleep apnoea, anemia, clinical characteristics, china, intracranial idiopathic hypertension

## Abstract

Introduction

Idiopathic intracranial hypertension (IIH) was previously assumed to be rare in Asia. However, it has been increasingly recognized in China in recent years, likely due to pandemic obesity and greater awareness of the condition. The clinical characteristics of IIH in Chinese patients remain unexplored. This study aims to investigate the clinical characteristics of patients with IIH in China.

Methods

A retrospective chart review of patients diagnosed with IIH at the First Affiliated Hospital of Zhengzhou University was conducted from January 2013 to July 2021. The analysis included demographic data, presenting symptoms, comorbidities, imaging features, laboratory data, intracranial pressure (ICP), treatment modalities, and outcomes.

Results

The study recruited 199 participants, including 145 females and 54 males, with a mean age at onset of 36 years (range: 27 to 45 years). The participants had a mean body mass index (BMI) of 26 kg/m2 (range: 23.4 to 29.4 kg/m2). Obesity was found in 67 participants (33.7%). The most common clinical symptom reported was headache, which was experienced by 118 (59.3%) participants, followed by decreased vision, which was reported by 115 (57.8%) participants. The main comorbidity among women was anemia (54, 37.2%), while men were more likely to have severe sleep apnea (7, 13%). The most common imaging features were perioptic nerve sheath distension (159, 79.9%) and transverse sinus stenosis (147, 73.9%). Symptoms were relieved with medication in 117 (58.8%) participants, while 72 (36.2%) underwent surgeries such as venous sinus stenting and ventriculoperitoneal shunt. During follow-up, symptoms resolved in 84 (42.2%) participants, while 115 (57.8%) participants experienced symptom improvement. The ratio of decreased vision was higher in females than in males.

Conclusion

The results provide valuable insights into the clinical features of IIH in this region. China appears to have a lower incidence of obesity compared to Western countries. Among comorbidities related to IIH, anemia and severe sleep apnea were the most common. A significant number of IIH patients underwent surgery. It was found that women had worse visual outcomes compared to men. Further investigation is needed to determine the most effective treatment for IIH in a larger cohort of Chinese patients.

## Introduction

Idiopathic intracranial hypertension (IIH) is characterized by elevated intracranial pressure (ICP) in the absence of any detectable cause [1]. The incidence rates of IIH vary across the world. In Western countries, the annual incidence of IIH is 12-20 per 100,000 people among obese women of childbearing age and 0.5-2 per 100,000 people in the general population [2-4]. However, IIH was believed to be relatively rare in Asia, with the annual incidence in Japan at only 0.03 per 100,000 people, much lower than in Western countries [5]. A retrospective study of IIH showed that only 12 Chinese patients were diagnosed with IIH in a 29-year period at a tertiary hospital in Taipei [6].

In recent years, IIH appears to be increasing in prevalence, not only due to the pandemic of obesity but also due to increased recognition of the disorder [7,8]. If not promptly recognized and managed, IIH can lead to severe visual impairment. There have been reports of Chinese IIH patients presenting with late-stage optic atrophy instead of papilledema [9]. We also found that some patients previously diagnosed as cerebral venous sinus thrombosis or optic neuritis may actually have IIH, indicating that IIH is not widely recognized by ophthalmologists and neurologists in China. Understanding the clinical characteristics of IIH is crucial for accurate diagnosis, appropriate treatment, and improved patient outcomes. Racial differences in the clinical features of IIH have been reported [10,11]; however, given the relatively low prevalence of IIH in our region previously, the clinical characteristics of IIH in China have not been fully investigated.

The objective of this study is to evaluate a cohort of patients diagnosed with IIH at a tertiary hospital in China over an 8.5-year period. This study will analyze patients’ demographic information, clinical features, comorbidities, treatment, and outcomes.

## Materials and methods

Ethical statement

This study was approved by the Ethics Committee of the First Affiliated Hospital of Zhengzhou University. 

Patient selection

A retrospective analysis was conducted on the medical records of patients diagnosed with IIH at the First Affiliated Hospital of Zhengzhou University in China from January 2013 to July 2021. The study protocol was approved by the Medical Ethics Committee of the First Affiliated Hospital of Zhengzhou University, with the approval number 2021-KY-1094. The data collected were anonymized, and the requirement for informed consent was therefore waived.

The inclusion criteria for the diagnosis of IIH are as follows: 1) papilledema confirmed on ophthalmoscopy, including secondary atrophy after papilledema; 2) normal neurological examination except for abducens nerve palsy; 3) normal neuroimaging findings with no evidence of hydrocephalus, mass, or structural lesions; 4) cerebrospinal fluid (CSF) open pressure measurement of ≥250 mmH_2_O in adults and ≥280 mmH_2_O in children; and 5) normal CSF constituents. The exclusion criteria included all other secondary causes of raised ICP. This study followed the tenets of the Helsinki agreement.

Demographic data and analyses

A standard protocol was used to conduct a retrospective chart review, which included demographic data, clinical characteristics, comorbidities, brain imaging, laboratory data, ICP, treatment modalities, and visual outcome. Demographic data involved recording the patients’ sex, age at onset, and body mass index (BMI), which was calculated as the body weight in kilograms divided by the square of the height in meters. Clinical characteristics included presenting symptoms, signs, ophthalmic and neurological examination. Detailed analysis of neuroimaging results, which included magnetic resonance imaging (MRI) and magnetic resonance venography (MRV) scans, were also recorded. Medical history, such as recent weight gain, anemia (hemoglobin < 12 g/dL), systemic hypertension, polycystic ovarian syndrome, sleep apnea, renal failure, etc., were also documented. Laboratory data consisted of ICP and the results of routine CSF examinations. Treatment regimen involved medical control and surgical intervention based on each individual case. Outcome was divided into two categories: “resolved,” which referred to the absence of headache, visual field defect, optic atrophy, or visual complaints during follow-up, and “improved,” which indicated the patient was headache-free but still had residual visual field defects or optic nerve damage. 

Statistical analysis

Descriptive statistics were used to summarize the data, and relevant associations were analyzed. The demographic characteristics of the patients were presented as means and percentages. Statistical analysis was performed using IBM SPSS Statistics for Windows, Version 23 (Released 2015; IBM Corp., Armonk, New York, United States). Measurement data with skewed distribution were expressed as means (P25, P75). Categorical data were expressed as rate (%), and comparison between groups was conducted using the χ2 test and Fisher exact test. All tests were two-sided, and a p-value less than 0.05 was considered statistically significant.

## Results

Demographic data

**Figure 1:**
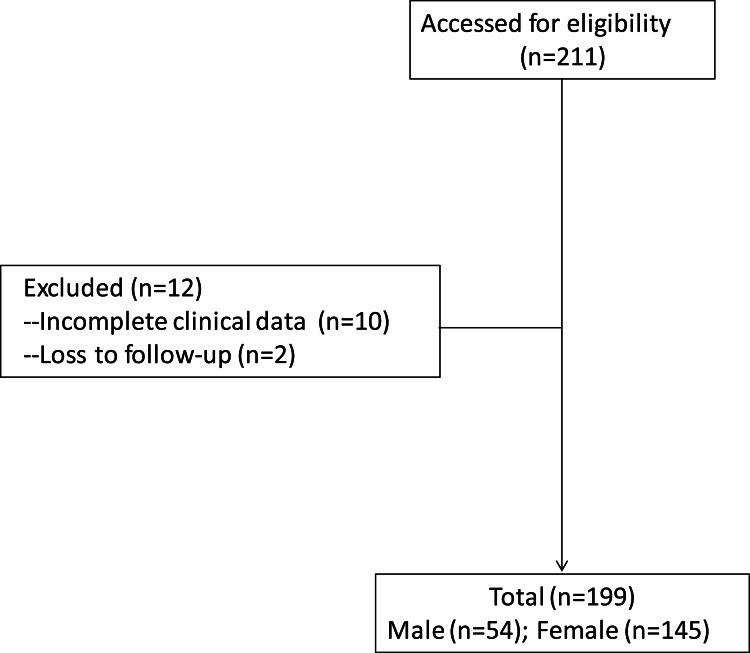
Flow diagram of the patient selection

Based on the medical records, 211 patients were diagnosed with IIH. Ten patients were excluded because of incomplete clinical data, and two patients were excluded from the analysis because of loss of follow-up. As a result, a total of 199 patients with IIH were included in the study (Figure 1). The majority of patients were female (145, 72.9%) and had a mean age of 44 (29, 49) years. The age at onset for men was 36 (22.5, 47) years old. The mean BMI was 26 (23.4, 29.4) kg/m^2^. For men, the mean BMI was 29.3 (26.3,29.75) kg/m^2^, and for women, it was 26 (25.7,28) kg/m^2^. Among the patients, 135 (67.8%) were overweight (28 kg/m^2 ^> BMI ≥ 24 kg/m^2^), and 67 (33.7%) were obese (BMI ≥ 28 kg/m^2^).

Clinical characteristics

The clinical characteristics of the patients in this study are as follows: 71 patients (35.7%) presented with acute onset of symptoms, 67 (33.7%) with subacute onset, and 61 (30%) with chronic onset. The most common presenting symptoms were headache (118 patients, 59.3%), decreased vision (115 patients, 57.8%), and nausea and vomiting (56 patients, 28.1%). Other chief complaints included dizziness (36 patients, 18.1%), tinnitus (27 patients, 13.6%), diplopia (23 patients, 11.6%), transient visual obscuration (17 patients, 8.5%), and seizure (five patients, 2.5%). Table 1 displays the detailed chief complaints of the IIH patients.

**Table 1:** Chief complaints of the IIH patients (n (%)) IIH: Idiopathic intracranial hypertension

Chief complaints	Total (n = 199)	Male (n = 54)	Female (n = 145)
Headache	118 (59.3%)	36 (66.7%)	83 (57.2%)
Decreased vision	115 (57.8%)	24 (44.4%)	91 (62.8%)
Nausea and vomiting	56 (28.1%)	14 (25.9%)	42 (29%)
Dizziness	36 (18.1%)	9 (16.7%)	27 (18.6%)
Tinnitus	27 (13.6%)	8 (14.8%)	19 (13.1%)
Diplopia	23 (11.6%)	5 (9.3%)	18 (12.4%)
Transient visual obscuration	17 (8.5%)	4 (7.4%)	13 (9%)
Seizure	5 (2.5%)	3 (5.6%)	2 (1.4%)

A range of medical conditions were found to coexist with IIH in this study. These included anemia in 59 patients (29.6%), endocrine abnormalities in 28 patients (14.1%), obstructive sleep apnea syndrome (OSA) in eight patients (4%), polycystic ovarian syndrome in three patients (1.5%), pregnancy in three patients (1.5%), recent weight gain in two patients (1%), and chronic renal dysfunction in two patients (1%). The comorbidities of the IIH patients are presented in Table 2. In comparison, among women, the primary comorbidity was anemia (54 patients, 37.2%) (p < 0.001), while OSA was the main comorbidity among men (seven patients, 13%) (p = 0.001).

**Table 2:** Comorbidities in the IIH patients (n (%)) IIH: Idiopathic intracranial hypertension

	Total (n = 199)	Male (n = 54)	Female (n = 145)	p-value
Anemia	59 (29.6%)	5 (9.3%)	54 (37.2%)	<0.001
Endocrine abnormalities	28 (14.1%)	6 (11.1%)	22 (15.2%)	0.464
OSA	8 (4%)	7 (13%)	1 (0.7%)	0.001
Polycystic ovarian syndrome	3 (1.5%)	0	3 (2%)	
Pregnancy	3 (1.5%)	0	3 (2%)	
Recent weight gain	2 (1%)	1 (1.9%)	1 (0.7%)	0.470
Chronic renal dysfunction	2 (1%)	1 (1.9%)	1 (0.7%)	0.470

Laboratory data and physical examination

As per the study’s findings, the average CSF opening pressure was found to be 350 (290, 400) mmH_2_O. Among males, the ICP averaged 335 (280, 400) mmH_2_O, while among females it was slightly higher at 360 (300, 400) mmH_2_O. Routine CSF examinations, including cell count, protein level, ordinary culture, were within the normal range. Papilledema was found in 121 patients (60.8%), with 24 (44.4%) males and 97 (66.9%) females displaying this symptom.

Neuroimaging results

All patients underwent MRI and MRV scan. The imaging results revealed that perioptic nerve sheath distension was the most commonly observed feature among patients, with 159 patients (79.9%) displaying this symptom. This was followed by transverse sinus stenosis, observed in 147 patients (73.9%), flattening of the globe in 129 patients (64.8%), tortuosity of the optic nerve in 102 patients (51.3%), empty sella in 86 patients (43.2%), optic disc protrusion in 53 patients (26.6%), and cerebellar tonsillar herniation in four patients (2.0%).The study also found that features such as empty sella (p＜0.001), optic disc protrusion (p＝0.021), and flattening of the globe (p＝0.019) were more common in females than in males. Table 3 provides further details regarding these findings.

**Table 3:** Imaging findings of the IIH patients n (%)) IIH: Idiopathic intracranial hypertension

	Total (n = 199)	Male (n = 54)	Female (n = 145)	p-value
Perioptic nerve sheath distension	159 (79.9%)	40 (78.4%)	119 (79.9%)	0.211
Transverse sinus stenosis (TSS)	147 (73.9%)	33 (61%)	114 (79%)	0.005
Ipsilateral TSS	87 (43.7%)	25 (46.3%)	62 (42.8%)	
Bilateral TSS	60 (30.2%)	8 (14.8%)	52 (35.9%)	
Flattening of the globe	129 (64.8%)	28 (51.9%)	101 (69.7%)	0.019
Tortuosity of the optic nerve	102 (51.3%)	25 (46.3%)	77 (53.1%)	0.393
Empty sella	86 (43.2%)	12 (22.2%)	74 (48.1%)	<0.001
Optic disc protrusion	53 (26.6%)	8 (14.8%)	45 (29.2%)	0.021
Cerebellar tonsillar herniation	4 (2.0%)	0	4 (2.8%)	0.576

Treatment and outcome

Various treatment modalities were employed. In 117 patients, medication (methazolamide and mannitol) was effective in relieving their symptoms. Overweight patients with a BMI ≥ 28 kg/m^2^ were encouraged to lose weight, while patients with anemia were advised to add iron supplements to their diet.

For the 72 (36.2%) patients who suffered from intractable loss of vision and persistent elevation of ICP despite medical treatment, surgery was recommended. Among them, 43 patients underwent venous sinus stenting, 16 patients had balloon dilatation, while six underwent combined balloon dilatation and stent placement. Three patients had a lumboperitoneal shunt, one of them underwent venous sinus stenting after lumboperitoneal shunt, and one underwent both a lumboperitoneal shunt and endoscopic repair of CSF rhinorrhea. Lastly, four patients underwent ventriculoperitoneal shunt, one of them after balloon dilatation failed.

During follow-up, it was found that the symptoms resolved in 84 (42.2%) of the patients, including 30 (55.6%) males and 54 (37.2%) females. In 115 (57.8%) patients, their symptoms improved, including 24 (44.4%) males and 91 (62.8%) females. It was also discovered that females were at a higher risk of decreased vision (p＝0.02). 

## Discussion

This study demonstrates several notable clinical characteristics of patients with IIH in China. The predominance of female patients and the high prevalence of overweight among this cohort aligns with previous findings from other geographic regions. However, the BMI of IIH patients in our study was relatively lower than those observed in Western countries, with a mean BMI of 26 kg/m^2^ present in our cohort of patients. In fact, differences in BMI between Asian and Western patients have been previously reported. It is worth noting that 65% to 75% of IIH patients in Western countries are obese [10,12-14]. In comparison, only 33.7% of IIH cases were found to be obese (BMI ≥ 28 kg/m^2^). These results are consistent with previous studies, indicating that ethnic differences may contribute to the pathogenesis of IIH. The findings also suggest that obesity may not be a significant risk factor for developing IIH in Asians [9,14].

The most common presenting symptoms, including headache, visual disturbances, and tinnitus, were well-established in the literature. The use of lumber puncture in the diagnostic workup and the characteristic neuroimaging findings provided additional evidence for the diagnosis of IIH. Comorbidities commonly co-occur with IIH [15]. In this study, we observed that anemia was the most frequent medical condition in our cohort of IIH patients, particularly in females. Most of the female IIH patients were obese and of childbearing age. Another Chinese study of IIH patients also noted that anemia was prevalent in pregnant women [16], possibly due to a high demand for iron during the perinatal period for fetal growth. Sleep apnea was the second most common underlying comorbidity observed in our study, especially in men, which is consistent with previous literature reports [9,17,18].

The management strategies employed in this cohort, including weight loss, medical therapy, and serial lumbar punctures, were in line with current treatment guidelines. However, a significant proportion of IIH patients underwent surgical interventions at our center. Surgical intervention was considered for patients with intractable vision loss and persistent elevation of ICP despite receiving medical treatment. Surgical procedures included venous sinus stenting, balloon dilatation, lumboperitoneal shunt, ventriculoperitoneal shunt, or a combination of these. Optic nerve sheath fenestration is rarely performed in China. The most effective treatment for IIH needs further investigation in a larger cohort of IIH patients.

The most severe complication of IIH is visual loss, with a high incidence (57.8%) of presentation with decreased vision observed in our cohort of IIH patients. During follow-up, a lower percentage of females showed a resolution of symptoms compared to males. Severe deficits in visual acuity have been reported in up to 25% of cases [19]. Results from our cohort study suggest that there is a higher risk of reduced vision in females residing in China.

The present study, conducted in a tertiary hospital in China, included only a relatively small number of IIH cases. Thus, the results obtained from this study may not be representative of the full extent of IIH in the general population. Future studies involving larger cohorts of IIH patients from multiple centers will be necessary to obtain a more comprehensive understanding of the condition.

## Conclusions

This study provides valuable insights into the clinical characteristics of patients with IIH China. There are racial differences in the demographic data of IIH in China, the obesity rate in this region may be lower than those in Western countries. Additionally, anemia and severe sleep apnea were the most common comorbidities among these patients. Early recognition and appropriate management of IIH are crucial in minimizing complications and preserving visual function. Further research is warranted to explore potential regional variations in IIH characteristics and to evaluate the long-term outcomes of different treatment modalities.

## References

[1] Markey KA, Mollan SP, Jensen RH, Sinclair AJ (2016). Understanding idiopathic intracranial hypertension: mechanisms, management, and future directions. Lancet Neurol.

[2] Raoof N, Sharrack B, Pepper IM, Hickman SJ (2011). The incidence and prevalence of idiopathic intracranial hypertension in Sheffield, UK. Eur J Neurol.

[3] Wall M (2008). Idiopathic intracranial hypertension (pseudotumor cerebri). Curr Neurol Neurosci Rep.

[4] Durcan FJ, Corbett JJ, Wall M (1988). The incidence of pseudotumor cerebri: population studies in Iowa and Louisiana. Arch Neurol.

[5] Yabe I, Moriwaka F, Notoya A, Ohtaki M, Tashiro K (2000). Incidence of idiopathic intracranial hypertension in Hokkaido, the northernmost island of Japan. J Neurol.

[6] Liu IH, Wang AG, Yen MY (2011). Idiopathic intracranial hypertension: clinical features in Chinese patients. Jpn J Ophthalmol.

[7] Mollan SP, Aguiar M, Evison F, Frew E, Sinclair AJ (2019). The expanding burden of idiopathic intracranial hypertension. Eye (Lond).

[8] Miah L, Strafford H, Fonferko-Shadrach B (2021). Incidence, prevalence and healthcare outcomes in idiopathic intracranial hypertension: a population study. Neurology.

[9] Chen Q, Feng C, Zhao G (2020). Pseudotumour cerebri syndrome in China: a cohort study. Sci Rep.

[10] Bruce BB, Preechawat P, Newman NJ, Lynn MJ, Biousse V (2008). Racial differences in idiopathic intracranial hypertension. Neurology.

[11] Wall M, Corbett JJ (2014). Revised diagnostic criteria for the pseudotumor cerebri syndrome in adults and children. Neurology.

[12] Sawada S, Sato Y, Aoyama H, Harada K, Nakanuma Y (2007). Pathological study of idiopathic portal hypertension with an emphasis on cause of death based on records of annuals of pathological autopsy cases in Japan. J Gastroenterol Hepatol.

[13] Qiao L, Wei Y (2020). Familial idiopathic intracranial hypertension in two non-obese Chinese sisters. Front Neurol.

[14] Kim TW, Choung HK, Khwarg SI, Hwang JM, Yang HJ (2008). Obesity may not be a risk factor for idiopathic intracranial hypertension in Asians. Eur J Neurol.

[15] Kilic K, Korsbæk JJ, Jensen RH, Cvetkovic VV (2022). Diagnosis of idiopathic intracranial hypertension-the importance of excluding secondary causes: a systematic review. Cephalalgia.

[16] Ma Z, Jiang H, Meng C, Cui S, Peng J, Wang J (2020). Idiopathic intracranial hypertension in patients with anemia: a retrospective observational study. PLoS One.

[17] Lee AG, Golnik K, Kardon R, Wall M, Eggenberger E, Yedavally S (2002). Sleep apnea and intracranial hypertension in men. Ophthalmology.

[18] Thurtell MJ, Trotti LM, Bixler EO (2013). Obstructive sleep apnea in idiopathic intracranial hypertension: comparison with matched population data. J Neurol.

[19] Binder DK, Horton JC, Lawton MT, McDermott MW (2004). Idiopathic intracranial hypertension. Neurosurgery.

